# Effects of the Oncoprotein PAX3-FOXO1 on Modulation of Exosomes Function and Protein Content: Implications on Oxidative Stress Protection and Enhanced Plasticity

**DOI:** 10.3389/fonc.2020.01784

**Published:** 2020-10-01

**Authors:** Assil Fahs, Farah Ramadan, Farah Ghamloush, Abeer J. Ayoub, Fatima Ali Ahmad, Firas Kobeissy, Yehia Mechref, Jingfu Zhao, Rui Zhu, Nader Hussein, Raya Saab, Sandra E. Ghayad

**Affiliations:** ^1^Department of Biology, Faculty of Science II, Lebanese University, Fanar, Lebanon; ^2^Department of Anatomy, Cell Biology and Physiology, American University of Beirut, Beirut, Lebanon; ^3^Department of Pediatrics and Adolescent Medicine, Children’s Cancer Institute, American University of Beirut, Beirut, Lebanon; ^4^Department of Biochemistry and Molecular Genetics, Faculty of Medicine, American University of Beirut, Beirut, Lebanon; ^5^Department of Chemistry and Biochemistry, Texas Tech University, Lubbock, TX, United States; ^6^Cancer Biology Stem Cells and Molecular Immunology Laboratory, Faculty of Sciences, Lebanese University, Beirut, Lebanon

**Keywords:** Rhabdomyosarcoma, PAX3-FOXO1, exosomes, oxidative stress, plasticity

## Abstract

Rhabdomyosarcoma (RMS) is a highly malignant soft tissue sarcoma classified into two major histologic subtypes: embryonal (ERMS) and alveolar (ARMS). ARMS subtype is clinically more aggressive, and characterized by an oncogenic fusion protein PAX3-FOXO1 (P3F) that drives oncogenic cellular properties. To understand the role of the fusion oncoprotein in paracrine signaling, we focused on secreted exosomes, which have been demonstrated to contribute to metastasis in multiple tumor types. Advanced Proteomics-bioinformatics analysis of the protein cargo of exosomes isolated from C2C12 myoblasts transduced with P3F fusion gene revealed 52 deregulated proteins compared to control cells, with 26 enriched and 26 depleted proteins. Using both PANTHER gene classification and Ingenuity Pathway Analysis (IPA) software, we found that the main biological processes in which the 52 deregulated proteins are involved, include “catalytic activity,” “binding,” “metabolic process,” and “cellular process.” The pathways engaging the 26 enriched proteins include the “14-3-3 mediated signaling,” “cell cycle,” and “ERK5, VEGF, IGF1,and p70S6K signaling.” Furthermore, the main nodes in which deregulated exosome proteins and miRNAs intersected revealed pathways conferring protection from stress and promoting plasticity. Based on the bioinformatics analysis and the altered exosome proteome profile, we performed biochemical functional analysis to study the diverse properties of these exosomes where angiogenesis, stemness, and anti-oxidative stress properties were validated using different platforms. P3F-modulated exosomes activated ERK, 4-EBP1, and MMP-2 in recipient cells, and enhanced angiogenesis and stemness. In addition, P3F led to lower cellular reactive oxygen species levels and enhanced resistance against oxidative stress; and treatment of stromal cells with P3F-modulated exosomes also conferred protection against exogenous oxidative stress. Our findings highlight the role of P3F fusion protein in modulating exosome cargo to confer a protective effect on recipient cells against oxidative stress and to promote plasticity and survival, potentially contributing to the known aggressive phenotype of the fusion gene-positive subtype of RMS.

## Introduction

Rhabdomyosarcoma (RMS) is an aggressive soft tissue sarcoma of the skeletal muscle generally affecting children and adolescents (1). It is currently classified, according to the World Health Organization (WHO), into four histological subtypes with embryonal (ERMS), and alveolar (ARMS) being the two most frequent subtypes (2). ARMS has been described as more aggressive having a prognostic disadvantage compared to ERMS, specifically in the majority subset expressing the fusion oncoprotein PAX3-FOXO1 (or less commonly PAX7-FOXO1) (3). In fact, fusion status which divides RMS cases into fusion positive (FPRMS) and fusion negative (FNRMS) allows a better classification and determination of prognostic features (4). Common translocations involve the DNA binding domain of either PAX3 or PAX7 with the transactivation domain of FOXO1 resulting in PAX3/7-FOXO1 fusion oncoprotein that enhances RMS growth and metastasis by acting at target genes involved in proliferation, migration and invasion (5). RMS cells can modulate the tumor microenvironment to enhance both cancer cell and recipient cell motility favoring metastatic disease (6). For instance, MMP (matrix metalloproteinase) production by RMS cells, and to a higher propensity by ARMS cells, has been found to induce extracellular matrix remodeling and enhance invasiveness (7).

Exosomes are extracellular vesicles with a diameter ranging between 20–150 nm secreted under both physiological and pathological conditions and their cargo modulates signaling pathways in recipient cells (8). They have been demonstrated to promote tumor growth in a variety of cancer types including Ewing sarcoma, osteosarcoma and melanoma by promoting metastasis (9–11). In fact, different proteins and nucleic acids are enriched or depleted within cancer cell-derived exosomes, an indication that their sorting into exosomes is a selective process and that cancer cells can influence recipient cell behavior through the release of exosomes with modulated cargo. Functional changes induced by cancer cell-derived exosomes include the activation of angiogenesis and enhancement of recipient cell migration and invasion (6, 12–15). Proteomic profiling of RMS-derived exosomes revealed the presence of proteins such as integrins and annexins in both FPRMS and FNRMS-derived exosomes which have been implicated in tumor metastasis (12, 13, 16, 17). Selective miRNA enrichment has also been demonstrated in RMS-derived exosomes and in P3F transduced myoblasts which were found to enhance recipient fibroblast migration and invasion (6, 18).

Cellular plasticity involves adaptation to changes in the microenvironment and physiological conditions that would allow cell survival and growth (19). In cancer, plasticity involves the acquisition of characteristics favoring tumor progression and cancer cell survival under stress conditions. For instance, Ewing sarcoma and melanoma cells were found to express the TGFβ co-receptor endoglin correlating with cell plasticity, enhanced survival and invasion (20). Plasticity also involves the attainment of stem cell characteristics that include an ability to invade and disseminate into surrounding tissue (21). Cancer stem cell (CSC) features have been observed in ERMS cells that express markers such as CD133, exhibit chemoresistance and can form rhabdomyospheres, all of which are stem cell characteristics (22, 23). Moreover, aspects of plasticity that sustain cancer cell survival include acquiring resistance to and protection from oxidative stress. Oxidative stress results from an imbalance between pro-oxidative and anti-oxidative cellular pathways. Reactive oxygen species (ROS) including hydrogen peroxide (H_2_O_2_), are required for physiological signal transduction, but their levels are elevated in cancer cells as a result of augmented metabolic rates (24, 25). While ROS can enhance cancer proliferation and metastasis, an over-accumulation of ROS creates oxidative stress that can threaten cell survival. Therefore, cancer cells exhibit resistance to oxidative damage by the activation of alternate signaling pathways with lower ROS production and increased induction of antioxidant enzymes (26). Of note, oxidative stress can also enhance exosomes production with altered nucleic acid content that can confer changes in recipient cells and sustain their survival against stress (27, 28). In this study, we demonstrate that the *P3F* fusion gene modulates exosomes to promote recipient cell plasticity and response to oxidative stress that may favor tumor growth and metastasis.

## Materials and Methods

### Cell Lines and Viruses

C2C12 mouse myoblasts were purchased from the ATCC (Virginia, United States) and cultured in Dulbecco’s modified Eagle’s medium AQ (DMEM AQ, Sigma, Setagaya, Japan) supplemented with 20% fetal bovine serum (FBS, Sigma) and 1% penicillin (100 units/ml) – streptomycin (100 μg/ml) antibiotics (Sigma). MSCV-IRES-GFP-PAX3-FOXO1 (MSCV-P3F) and MSCV-IRES-GFP (MSCV-GFP) plasmids were a kind gift from Dr. Gerard Grosveld (St. Jude Children’s Research Hospital, Memphis, United States). The human ERMS cell lines JR1 and the ARMS cell lines Rh30, were generously donated by Dr. Peter Houghton (Columbus, OH, United States), and have been previously described [reviewed in (29)]. HUVEC (human umbilical vein endothelial cells) and HEK293T (human embryonic kidney) cells were also purchased from ATCC. As described previously, viral supernatants were produced by transfecting 293T cells with MSCV-P3F or MSCV-GFP vectors using calcium phosphate (18). C2C12 cells were then transduced in suspension with either MSCV-GFP viruses (forming Ctrl-C2C12 cells) or MSCV-P3F (forming P3F-C2C12 cells) at 32°C, 1250 × *g* for 1 h with 8 μg/ml Polybrene (hexadimethrine bromide; Sigma), and sorted using FACS Aria SORP cell sorter (BD, New Jersey, United States) after selection with 2 μg/ml Puromycin (Abcam). Wild-type mouse embryonic fibroblasts (MEFs) and *p53−/−* (FVB.129-*Trp53*^tm1Brn^) MEFs were isolated from E13.5 embryos of mixed C57BL/6 × 129/Sv 77 background (Jackson Laboratory, Maine, United States) using the procedure approved by the Institutional Care and Use Committee (IACUC) at the American University of Beirut, and following the IACUC-approved guidelines. MEFs are cultured in DMEM AQ, media supplemented with 10% fetal bovine serum (FBS, Sigma), 1% penicillin (100 units/ml) – streptomycin (100 μg/ml) antibiotics (Sigma), 1% sodium pyruvate, and 1% non-essential amino acids. All cells were maintained under standard conditions (humidified atmosphere, 95% air, 5% CO2, 37°C).

### Exosome Isolation

Exosomes were isolated as previously described (6). Briefly, Ctrl-C2C12 cells, P3F-C2C12 cells, JR1 and Rh30 cells were incubated in exosome-free medium prepared by ultracentrifugation at 100,000 × *g*, overnight at 4°C. Exosomes were then isolated from the conditioned media by differential ultracentrifugations (18): 300 × *g* for 10-min 2,000 × *g* for 20-min centrifugation, and then 10,000 × *g* for 30 min. Finally, ExoQuick Exosome Precipitation Solution (System Biosciences, mountainite, CA, United States) was added to the resulting supernatant, and stored overnight at 4°C to allow exosome precipitation. The following day, the condensed medium was ultracentrifuged at 100,000 × *g* for 70 min, the pellet resuspended in PBS 1X, and ultracentrifuged at 110,000 × *g* for 70 min to remove any contaminating element. The final pellet was resuspended in 300 μL of PBS for functional assays and stored at −80°C. All centrifugations were done at 4°C. Recipient cells were treated with exosomes at 1X and 10X concentration, where 1X corresponds to exosomes isolated from an equivalent number of cells to those treated and 10X is 10 times this value.

### Protein Extraction, and Western Blot

Proteins were extracted from 3 independently transduced cells and their respective exosomes using CHAPS lysis buffer (30 mM Tris–Cl, pH 7.5; 150 mM NaCl; and 1% CHAPS) mixed with 25X protease inhibitor (Roche, Basel, Switzerland). After adding the appropriate volume of lysis buffer, the mixture was sonicated for 20 min, centrifuged for 30 min at 13,000 × *g* at 4°C, then the supernatant containing the proteins was collected (12) and quantified using Bradford protein assay. Equal amounts of proteins were loaded and separated using 10% or 12% acrylamide gels, then transfered to a polyvinylidene difluoride (PVDF, Bio-Rad, CA, United States) membrane or nitrocellulose membrane (Santa Cruz, Heidelberg, Germany) in TGS1X-10% methanol transfer buffer. Membranes were blocked to prevent non-specific binding with 3% BSA-TBS1X-0.001% Tween [Tris (hydroxymethyl); NaCl; KCl and Tween 20; pH = 7.5] or 5% milk-TBS1X-0.001% Tween for 1 h, and then probed with primary antibodies (listed below) at 4°C overnight where they were washed 3 times by TBS1X-0.001% Tween for 5 min before adding the corresponding species-specific Horseradish Peroxidase (HRP)-conjugated secondary antibodies (Santa Cruz) for 2 h. Results were then detected on ChemiDoc machine (Bio-Rad) using Clarity Western ECL reagent (Bio-Rad) as a substrate.

The primary antibodies used were: anti-Hsc70, anti-GAPDH, anti-Vimentin, anti-Fibronectin, anti-Actin, anti-Basigin, anti-Calnexin (Santa Cruz), anti-phospho-ERK, anti-phospho-4EBP1, anti-ERK, anti-4EBP1 (Cell signaling, Danvers, United States), anti-TSG101 (Abcam, Cambridge, United Kingdom), anti-gp47, and anti-gp91, and anti-NOX1 (Upstate, NY, United States).

### LC-MS/MS Analysis

Extracted proteins from Ctrl-C2C12- and P3F-C2C12-derived exosomes were subjected to sample clean-up and tryptic digestion as described previously (12). The LC-MS analysis of the tryptic digested proteins was performed using a 3000 Ultimate nano-LC system (Dionex, CA, United States) interfaced to an electrospray ionization (ESI) source equipped LTQ Orbitrap Velos mass spectrometer (Thermo Scientific, CA, United States). Aliquots of 1 μg of tryptic digests were injected and initially desalted on-line using an Acclaim PepMap100 C18 pre-column (75 μm I.D. 20 mm length, 3 μm particle sizes, 100Å particle size, Thermo Scientific) at a flow rate of 3 μL/min. The purified peptides were separated using an Acclaim PepMap100 C18 capillary column (75 μm I.D., 150 mm length, 2 μm, 100Å, Thermo Scientific) at a flow rate of 0.35 μL/min. The gradient applied to achieve separation was as follow: solvent B (99.9% ACN with 0.1% formic acid) was maintained at 5% from 0 to 10 min, increased to 20% over 55 min, 20–30% over 25 min, 30–50% over 20 min, 50–80% over 1 min, 80% over 4 min, decreased to 5% over 1 min, and finally was maintained at 5% for 4 min. Solvent A was 98% HPLC water, 1.9% ACN, and 0.1% FA. The LTQ Orbitrap Velos was operated in a data-dependent acquisition mode with 2 scan events. The first scan was a full MS scan of m/z 400-2000 with a mass resolution of 15,000. The second scan event was a CID MS/MS repeated on top 10 intense ions selected from the previous scan event with an isolation window of m/z 3.0. The mass spectrometry proteomics data have been deposited to the ProteomeXchange Consortium via the PRIDE partner repository.

### LC-MS Data Processing

Proteome Discover version m1.2 (Thermo Scientific) was used to convert raw files acquired from LC-MS analysis to mascot generic format (^∗^.mgf) files. The ^∗^.mgf files were subjected to MASCOT version 2.4 (Matrix Science Inc., Boston, United States) to search against protein database. The carbamidomethylation of cysteine was a fixed modification and the oxidation of methionine was considered as a variable modification. The mass shift tolerance for matching peptide precursors and fragments was 6 ppm and 0.8 Da, respectively. Enzyme was specified as trypsin. A maximum of 2 miscleavage was allowed. MASCOT searching results were then exported to Scaffold (version 3.6.3, Proteome Software, Portland, OR, United States) for further processing and quantification. Identified proteins were filtered using the following criteria: Peptide threshold = 95%, protein thresholds = 99%, with a minimum of 2 peptides identified. The quantification was based on spectral counting. The quantitative proteomic results were further subjected to statistical analysis.

### Bioinformatic Analysis

For gene ontology analysis, including differential molecular function and biological processes, PANTHER software (Protein Analysis Through Evolutionary Relationships; http://www.pantherdb.org/genes/batchIdSearch.jsp) was used to classify genes into distinct categories of molecular functions and biological processes. Genes were classified into families and subfamilies of shared function, which were then categorized using a highly controlled vocabulary (ontology terms) by the biological process and molecular function. Functional pathways and protein association were examined using the computational bioinformatics program, Pathway Studio software (v.11.4.0.8; Elsevier) as previously described (30, 31). This bioinformatics platform enables the analysis and visualization of altered pathways and protein network association needed to construct and recognize altered cellular processes and involved molecular functional pathways.

The identified proteins were analyzed using Ingenuity Pathway Analysis (IPA) software (Ingenuity^®^ Systems, www.ingenuity.com), to find diseases, functions, and networks in which the identified proteins are implicated; the analysis was done for the deregulated proteins with their fold changes. The analysis was also done by PANTHER Software^1^, which was used to identify the molecular processes and biological pathways linked to these proteins.

### Gelatin Zymography Assay

Proteins were extracted from culture supernatant of MEF cells treated with 10X exosomes for 72 h and run along with FBS (positive control) on 0.75 mm SDS-PAGE gel, containing gelatin (Bio-Rad) as a substrate, washed twice and incubated overnight in substrate buffer at 37°C with shaking. Gels were then stained with coomassie blue (Thermo Scientific) for 1 h at RT, de-stained with ethanol, acetic acid and water solution. Results were detected on ChemiDoc machine. Band staining intensity was determined by densitometry, using ImageJ software (32).

### Tube Formation Assay

Matrigel (Corning, NY, United States) was diluted 1:1 by HUVEC cell medium and added very carefully into a 96-well plate, avoiding bubbles. The plate was placed in the incubator at 37°C for 30 min to solidify, after which HUVEC cells (15,000 cells/well) were plated on top and treated with either Ctrl-C2C12-derived exosomes or P3F-C2C12-derived exosomes at1X and 10X concentrations then left for 2 h to form tubes. Images were taken by light transmission microscopy and analyzed by ImageJ software (angiogenesis analyzer) as previously performed (6).

### Sphere Formation Assay

*p53−/−* MEFs and C2C12 cells (2 × 10^3^/well) suspended in Matrigel^TM^ :serum-free media (1:1) were uniformly plated around the bottom rim of 24-well plates with 3% serum-containing media supplemented with 20 ng/mL epidermal growth factor (EGF, R&D Systems, Minnesota, United States) and 2% B27 (Invitrogen, CA, United States). The medium was replenished every 72 h. Micrographs of the spheres formed after 5 days were taken and counted (33).

### MTT Proliferation Assay

Transduced C2C12 cells (8000 cell/well) were plated in 96-well plates. The following day, they were treated with different concentrations of H_2_O_2_ (Sigma) ranging from 0 to 500 μM. MTT (Roche) was added according to the manufacturer’s instructions after 24 h. The absorbance was measured by ELISA reader (at a 595 nm wavelength). Results were computed as the mean percent absorbance of H_2_O_2_-treated condition relative to control (untreated). Each experiment was repeated at least 3 times, each performed in triplicate.

### Trypan Blue Exclusion Assay

C2C12 cells were seeded in 24-well plates at a density of 10,000 cells/well. After 5 h of incubation at 37°C, Ctrl- C2C12-, or P3F-C2C12-derived exosomes (at 10X concentration) were added to the cells. Then 40 h later, cells were treated simultaneously with 60 μM H_2_O_2_ and Ctrl-C2C12 or P3F-C2C12 exosomes at 10X concentration. Cells were counted using trypan blue (Sigma) at 8 and 24 h following H_2_O_2_ treatment. Each experiment was repeated at least 3 times, each performed in triplicate.

### Flow Cytometry for ROS Production Evaluation

Reactive oxygen species levels were evaluated for Ctrl-C2C12 and P3F-C2C12, or C2C12 cells treated with Ctrl-C2C12-derived exosomes or P3F-C2C12-derived exosomes at a 10X concentration. In summary, 30,000 cells were seeded in 6-well plates, and incubated at 37°C for 48 h. When needed, exosomes were added 5 h post plating. On the day of the experiment, control wells were incubated for 1 h with either H_2_O_2_ (positive control) or 10 mM N-Acetyl-Cysteine (NAC), negative control, which is a ROS inhibitor (Sigma). ROS was detected by incubating cells for 30 min with the cell permanent reagent 2’, 7’–dichlorofluorescin diacetate (DCF-DA; 1 mg/ml, Sigma), a fluorogenic dye that measures hydroxyl, peroxyl, and other ROS activity within the cell. After diffusion into the cell, DCF-DA is deacetylated by cellular esterases to a non-fluorescent compound, which is later oxidized by ROS into 2’, 7’ –dichlorofluorescein (DCF). DCF is a highly fluorescent compound, which can be detected by fluorescence spectroscopy with maximum excitation and emission spectra of 495 nm and 529 nm, respectively. Cells were then trypsinized, centrifuged at 1500 rpm for 7 min, and the pellet was resuspended with 300 μL of PBS. Finally, cells were analyzed by the Guava EasyCyte8 flow cytometer.

### Statistical Analysis

Statistical comparisons were made between the control and treatment groups using Student’s *t*-test. A *p*-value < 0.05 was considered to indicate a statistically significant difference. The PCA analysis for LC-MS results was performed by MarkerView 1.1 (AB Sciex, Framingham, United States).

## Results

### *PAX3-FOXO1* Alters Protein Cargo of C2C12 Derived Exosomes

**FIGURE 1:**
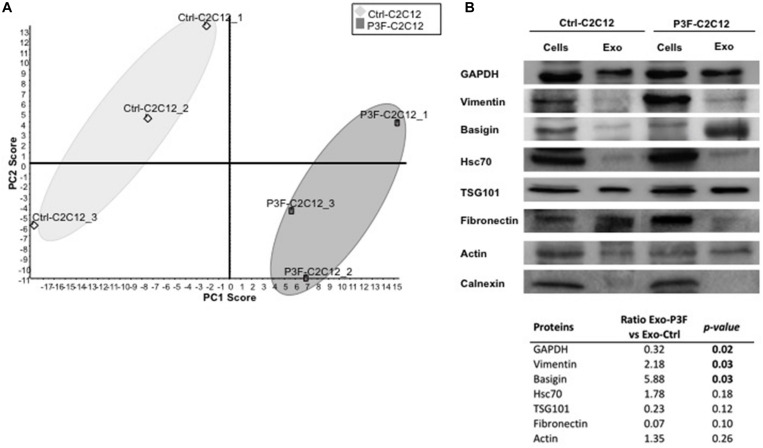
LC-MS/MS protein profiling of C2C12-derived exosomes. **(A)** Score plot obtained by Principal Component analysis (PCA) for LC–MS/MS results of 3 independently transduced preparations of Ctrl-C2C12 and P3F-C2C12 exosomes. **(B)** Western Blot analysis of the indicated proteins in exosomes and respective cell lysates. The table represents the expression level of the represented proteins in P3F-C2C12 exosomes compared to Ctrl-C2C12 exosomes as quantified by LC-MS/MS with their respective *p*-values.

The myogenic background of the murine C2C12 myoblasts is the ideal system to evaluate cellular effects of *P3F* (18, 34). As previously published, C2C12 cells transduced with P3F-expressing vector (P3F-C2C12) show features of transformation, including enhanced anchorage-independent growth when compared to cells transduced with empty vector (Ctrl-C2C12) (18). Analysis of this system previously showed that *P3F* alters the miRNA content of exosomes, leading to over-expression of *miR-486-5p*, in turn promoting pro-tumorigenic cellular properties (18). However, the effect of possible changes in the protein content of exosomes in response to P3F has not been characterized to date. In this study, we interrogated the proteomic content of C2C12 exosomes in response to P3F expression. We extracted exosomal proteins and subjected them to LC-MS/MS proteomics analysis (Supplementary Figure 1). The results showed excellent reproducibility within each cell line (Figure 1A). Principal components analysis (PCA) shows that the protein content of 3 independent extractions from Ctrl-C2C12 exosomes clustered together and separately from the 3 independent protein extractions from P3F-C2C12 exosomes, meaning that there is a difference between the protein cargo of Ctrl-C2C12 and P3F-C2C12 exosomes (Figure 1A). LC-MS/MS results were validated by western blot for a subset of proteins identified to be common among Ctrl-C2C12 and P3F-C2C12 exosomes, showing excellent concordance of results. Along with the common exosomal protein markers, TSG101, Hsc70, and GAPDH, we detected the presence of Vimentin, Basigin, Fibronectin, and Actin. The negative control Calnexin, a cellular protein that is localized to the endoplasmic reticulum was, as expected, excluded from exosomes (Figure 1B).

By comparing the expression levels of the proteins found within P3F-C2C12-derived exosomes to those within Ctrl-C2C12-derived exosomes revealed by LC-MS/MS analysis, we identified 52 proteins differentially expressed; 26 proteins were significantly enriched in P3F-C2C12-derived exosomes among which 7 were exclusively present within these exosomes and 26 were significantly down-regulated proteins among which 13 were excluded from these exosomes (Table 1).

**TABLE 1:** List of the 26 significantly enriched and 26 significantly down-regulated proteins in P3F-C2C12-derived exosomes compared to those in Ctrl-C2C12-derived exosomes with their fold changes and *p*-value.

		Expression level Exo-P3F *versus* Exo-Ctrl	*p*-value
	**Enriched proteins**		
1	Leukocyte elastase inhibitor (Serpinb1a)	Infinity	6.3.E-06
2	Lymphocyte antigen 6C1 (Ly6c1)	Infinity	7.5.E-04
3	Lymphocyte antigen 6D (Ly6d)	Infinity	2.6.E-03
4	Histone H1.5 (Hist1h1b)	Infinity	7.0.E-03
5	Calreticulin (Calr)	Infinity	8.9.E-03
6	Retinoic acid-induced protein 3 (Gprc5a)	Infinity	2.8.E-02
7	Rho GDP-dissociation inhibitor 2 (Arhgdib)	Infinity	3.7.E-02
8	Prostaglandin reductase 1 (Ptgr1)	28.2	2.9.E-03
9	Embigin (Emb)	15.47	2.0.E-02
10	Basigin (Bsg)	5.88	3.3.E-02
11	Hsc70-interacting protein (St13)	3.74	2.3.E-02
12	Glutathione S-transferase P 1 (Gstp1)	3.65	2.2.E-02
13	Nucleoside diphosphate kinase B (Nme2)	3.56	3.8.E-02
14	Annexin A1 (Anxa1)	3.49	4.6.E-03
15	Superoxide dismutase (Sod1)	3.45	3.4.E-04
16	Monocarboxylate transporter 1 (Slc16a1)	3.41	2.3.E-02
17	Granulins (Grn)	2.94	3.3.E-02
18	Fatty acid-binding protein, epidermal (Fabp5)	2.76	7.4.E-03
19	Alpha-actinin-1 (Actn1)	2.68	1.7.E-02
20	14-3-3 protein zeta/delta (Ywhaz)	2.2	9.1.E-04
21	Vimentin (Vim)	2.18	3.5.E-02
22	CD82 antigen (Cd82)	2.06	9.7.E-03
23	Major vault protein (Mvp)	2.01	9.0.E-03
24	Malate dehydrogenase, cytoplasmic (Mdh1)	1.84	3.9.E-02
25	14-3-3 protein epsilon (Ywhae)	1.82	4.7.E-02
26	Sodium/potassium-transporting ATPase subunit alpha-1 (Atp1a1)	1.7	3.2.E-02
	**Down-regulated proteins**		
1	Alpha-enolase (Eno1)	0.67	4.5.E-02
2	Glyceraldehyde-3-phosphate dehydrogenase (Gapdh)	0.32	1.5.E-02
3	Adenosylhomocysteinase (Ahcy)	0.28	4.8.E-02
4	T-complex protein 1 subunit theta (Cct8)	0.23	4.9.E-02
5	T-complex protein 1 subunit zeta (Cct6a)	0.21	2.7.E-02
6	Integrin alpha-5 (Itga5)	0.2	2.2.E-02
7	Serine protease HTRA1 (Htra1)	0.19	4.0.E-02
8	Myosin-9 (Myh9)	0.18	2.8.E-02
9	Voltage-dependent calcium channel subunit alpha-2/delta-1 (Cacna2d1)	0.14	3.9.E-02
10	Transitional endoplasmic reticulum ATPase (Vcp)	0.13	2.8.E-02
11	Talin-1 (Tln1)	0.13	5.7.E-03
12	Peptidyl-prolyl *cis*-trans isomerase C (Ppic)	0.08	7.2.E-03
13	Filamin-A (Flna)	0.02	1.0.E-03
14	Sorcin (Sri)	0	4.3.E-06
15	Serine protease 23 (Prss23)	0	4.7.E-04
16	Ras-related protein Rab-5C (Rab5c)	0	4.7.E-04
17	N(G),N(G)-dimethylarginine dimethylaminohydrolase 2 (Ddah2)	0	9.5.E-04
18	Mimecan (Ogn)	0	4.7.E-03
19	Band 4.1-like protein 2 (Epb41l2)	0	1.1.E-02
20	Ras-related protein R-Ras2 (Rras2)	0	1.4.E-02
21	EGF-like repeat and discoidin I-like domain-containing protein 3 (Edil3)	0	2.4.E-02
22	Inter-alpha-trypsin inhibitor heavy chain H3 (Itih3)	0	2.4.E-02
23	DnaJ homolog subfamily A member 2 (Dnaja2)	0	2.9.E-02
24	LIM and senescent cell antigen-like-containing domain protein 1 (Lims1)	0	3.4.E-02
25	Large neutral amino acids transporter small subunit 1 (Slc7a5)	0	3.4.E-02
26	Integrin-linked protein kinase (Ilk)	0	3.8.E-02

*The expression levels presented are the ratio of the level of the indicated proteins found by LC-MS/MS within the P3F-C2C12 exosomes compared to Ctrl-C2C12 exosomes.*

### PAX3-FOXO1 Fusion Protein Modifies the Exosomal Protein Cargo in Favor of Processes and Pathways Implicated in Cancer Progression

**FIGURE 2:**
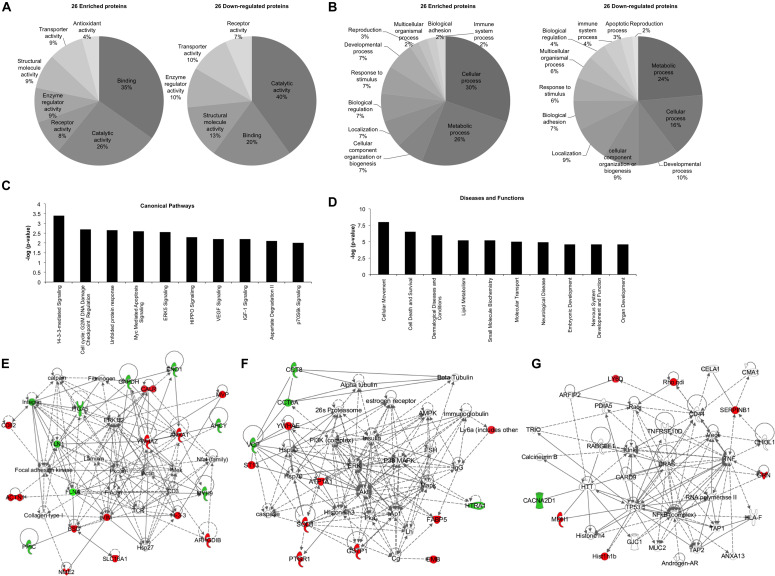
Bioinformatics analysis of the deregulated proteins between Ctrl-C2C12 and P3F-C2C12 exosomes. **(A,B)** Pie charts determined by Panther analysis presenting the molecular functions **(A)**, and the biological processes **(B)** for the 26 enriched and 26 down-regulated proteins. **(C,D)** histograms from IPA analysis of the 26 enriched proteins showing the top 10 canonical pathways **(C)** and the top 10 diseases and functions **(D)**. **(E–G)** Generated networks by IPA analysis of the deregulated proteins in C2C12-P3F-derived exosomes compared to Ctrl-C2C12-derived exosomes. Proteins colored in red are enriched and those colored in green are down-regulated. The higher the level of enrichment or down-regulation, the more intense the color is presented. Edges (lines and arrows between nodes) represent direct (solid lines) and indirect (dashed lines) interactions between molecules as supported by information in the Ingenuity knowledge base.

Bioinformatics analysis was performed in order to unravel the possible effects that the 52 deregulated proteins can impose on recipient cells, using the two software: Panther classification system and IPA. Panther software highlighted the molecular functions and the biological processes in which these 52 deregulated proteins were implicated (Figures 2A,B). “Catalytic activity” and “binding” were the top molecular functions for both enriched and down-regulated proteins (Figure 2A). It is important to note that “antioxidant activity” is the only molecular function exclusively found in the exosomes derived from P3F-C2C12 cells with 4% of the enriched proteins implicated (Figure 2A). Furthermore, “metabolic process,” and “cellular process” were the top biological processes in which both enriched and down-regulated proteins are implicated (Figure 2B). “Apoptotic process” appears to be the only biological process eliminated from the exosomes after P3F transduction (Figure 2B). These processes underline the implication of the P3F-C2C12-derived exosomes in tumor progression.

Ingenuity Pathway Analysis software was used to assess the canonical pathways implicated and the downstream diseases and functions in which the 26 significantly enriched proteins are involved (Figures 2C,D). The enrichment of the identified proteins seem to implicate “14-3-3 mediated signaling,” “cell cycle: G2/M DNA damage checkpoint regulation,” and “ERK5, VEGF, IGF1, and p70S6K signaling,” all of which are important in cell plasticity and cancer progression (Figure 2C). Moreover, “cellular movement” and “cell death and survival” appear in the top 10 diseases and functions where these deregulated proteins are implicated (Figure 2D). IPA software allows for identification of common networks in which the deregulated proteins may be intersecting. Three networks resulted from this analysis with all three having more than one identified deregulated focus molecule (Figures 2E–G). In these networks, we could identify important nodes previously implicated in RMS biology and progression such as: FAK, PKC, MEK, PI3K/AKT/ERK/MAPK, NFkB, TP53, HRAS, as well as proteins involved in cellular signaling and angiogenesis such as VEGF.

### PAX3-FOXO1-Deregulated Proteins and miRNA in Exosomes Are Involved in Cell Survival and Cellular Plasticity

**FIGURE 3:**
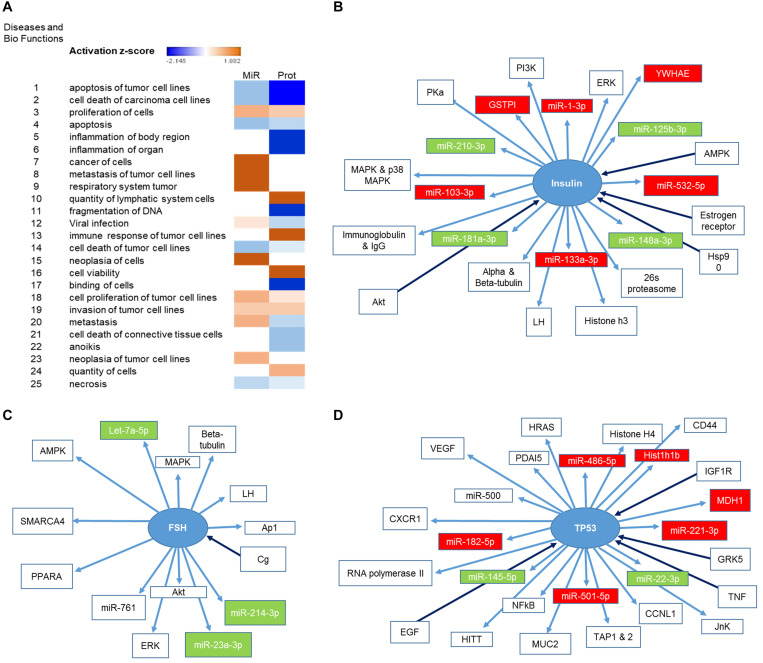
Identification of the pathways in which both deregulated miRNA and proteins are implicated. **(A)** Heat map representing the diseases and bio functions in which both deregulated miRNA and proteins are implicated and their corresponding activation score. **(B,D)** Representation of the upstream and downstream miRNA and proteins in pathways centering on **(B)** Insulin, **(C)** FSH, and **(D)** TP53. Proteins and miRNAs colored in red are enriched and those colored in green are down-regulated.

We had previously characterized the miRNA cargo modulated by P3F in C2C12-derived exosomes (18). We now paired both the deregulated 111 miRNA and the 52 proteins using IPA, and found largely common functions where these molecules are implicated, specifically down-regulating apoptosis, cell death and necrosis, and inducing cell proliferation, invasion, and metastasis (Figure 3A). This result is in line with the functional effect of P3F-C2C12 exosomes presented in our previous work (18), where these exosomes were found to promote viability, invasion, and migration of recipient cells. Comparing the generated networks by IPA from miRNA and proteins separately, we identified 3 common nodes focusing on Insulin, Follicle-stimulating hormone (FSH), and TP53. Figures 3B–D clarify the upstream and downstream deregulated miRNA and proteins identified within the exosomes that are regulated by or regulate these three nodal proteins.

### PAX3-FOXO1-Modulated Exosomes Induce Cell Survival Pathways, Angiogenesis and Stemness in Recipient Cells

**FIGURE 4:**
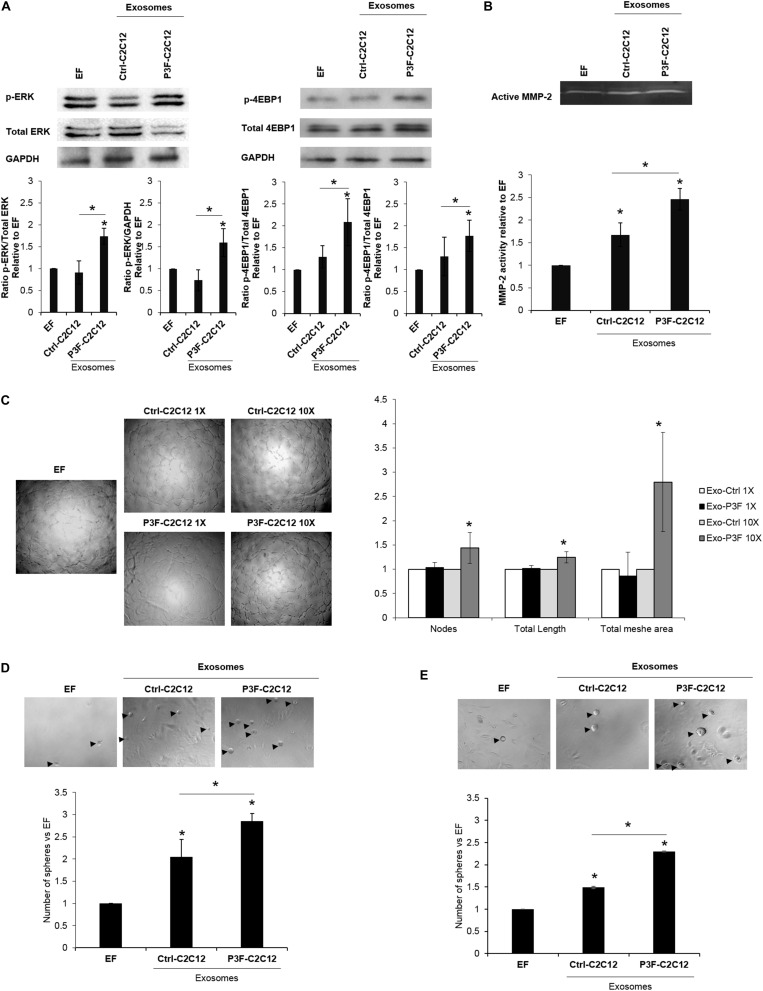
C2C12-P3F-derived exosomes promote angiogenesis and induce stemness of recipient cells. **(A)** Western blot for the indicated proteins in MEFs treated with Exo-free (EF) media, 10X Ctrl-C2C12-derived exosomes, and 10X P3F-C2C12-derived exosomes. GAPDH is used as a loading control. Histograms presenting the levels of phosphorylation of ERK and 4EBP1 compared to Exo-free condition, quantified by ImageJ software from *n* = 3 different western blots. **(B)** Zymography image of digested regions showing MMP2 activity in MEFs treated with either Ctrl-C2C12-derived exosomes or P3F-C2C12-derived exosomes for 72 h. Relative MMP-2 activity from 3 independent experiments was quantified by ImageJ software and presented by a histogram. **(C)** Representative phase contrast photomicrographs of endothelial tube formation of HUVECs cultured on Matrigel with either Exo-Free medium (EF), 1X, or 10X C2C12-MSCV-derived exosomes or C2C12-P3F-derived exosomes, as specified. Quantitation of the total number of nodes, total length and total mesh area of the different conditions are shown in the histogram, which represent the mean of 3 independent experiments, each performed in triplicate. **(D,E)** Representative phase contrast photomicrographs and histograms showing the ratio of the spheres formed by p53−/− MEFs **(D)** or C2C12 cells **(E)** treated with either Exo-Free medium (EF) or 10X C2C12-MSCV-derived exosomes or C2C12-P3F-derived exosomes, as specified. All data are reported as means of at least three independent experiments ± SD. Asterisks (*) denote a statistically significant difference (*p*-value < 0.05).

We have previously shown that exosomes isolated from P3F-C2C12 cells were able to induce proliferation, invasion and migration of recipient cells (MEFs and C2C12) compared to cells treated with Ctrl-C2C12-derived exosomes (18). In line with the pathways identified by network analysis in Figures 2C,E,F, we verified that treatment with P3F-C2C12-derived exosomes leads to increased phosphorylation (and therefore activation) of ERK and 4-EBP1 (Figure 4A), which are essential mediators of cancer cell survival and proliferation (35, 36).

In view of the nodal proteins identified to be involved in cytoskeletal organization and matrix degradation, such as Basigin, Filamin A, and others (Figure 2E), we examined the functional effects of the modulated exosomes on collagenase activity using zymography assay. Indeed, P3F-modulated exosomes led to higher MMP-2 activity in treated MEFs, as compared to cells treated with Ctrl-C2C12-derived exosomes evident by the increase in gelatinolytic bands (digested regions; Figure 4B).

Since the identified networks also involved angiogenic proteins such as VEGF and TNF (Figures 2C,G), we investigated the effect of P3F-C2C12-derived exosomes on angiogenesis by conducting a matrigel tube formation assay that evaluates the ability of HUVECs to differentiate into capillary-like structures when plated on matrigel (6). Counting the nodes, which are defined as the intersection of 3 or more branches of tubular connections, the total length which is the sum of length of segments, isolated elements and branches in the analyzed area and the total mesh area, which represents the area occupied by the primary capillary-like tubes, we found a significant increase when HUVECs were treated with P3F-C2C12-derived exosomes compared to control, at 10X but not 1X concentration (Figure 4C).

Exosomes can reprogram transcription within recipient cancer cells allowing their dedifferentiation into CSC with sphere-forming capacities (37). To examine whether P3F-modulated exosomes would enhance their ability to confer stem-like properties to recipient cells, we examined their effects on sphere-forming capacity of MEFs and C2C12 cells. We used *p53−/−* MEFs for this assay, as wild-type MEFs were not prone to forming spheres in culture. For both *p53−/−* MEFs and C2C12 cells, we found that treatment with P3F-modulated exosomes led to a significant increase in the number of formed spheres 5 days post treatment, relative to the control-derived exosomes (Figures 4D,E). These results highlight the ability of the P3F fusion protein to promote cellular plasticity and tumorigenic aspects through exosomes.

### PAX3-FOXO1 Fusion Protein Is Implicated in Redox Homeostasis and Tolerance/Protection From Oxidative Stress by Decreasing ROS Levels in Both Transduced and Recipient Cells

**FIGURE 5:**
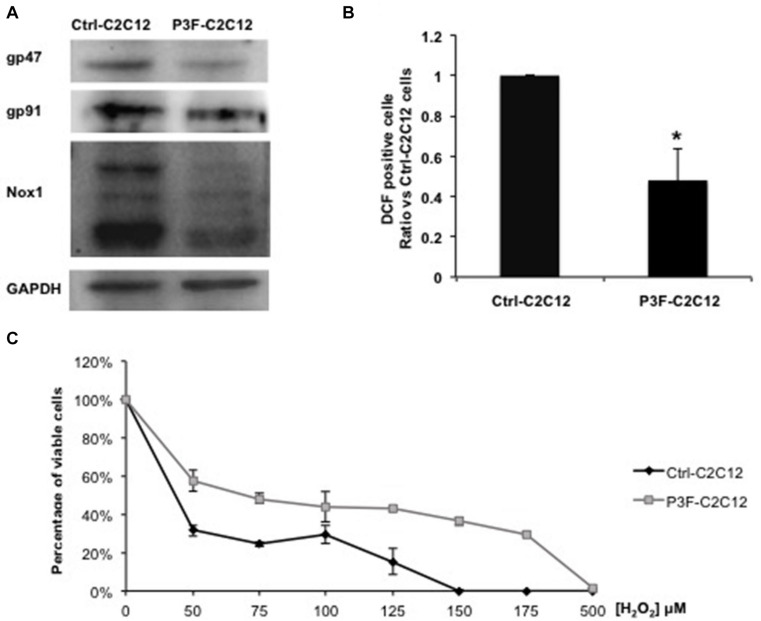
P3F decreases ROS levels in transduced cells. **(A)** Western blot analysis of gp47, gp91, and Nox1 protein levels; subunits of the NADPH oxidase complex. GAPDH is used as a loading control. **(B)** Histogram representing the percentage ± SD of DCF-DA positive cells, relative to control Ctrl-C2C12 cells; numbers shown are average of three independent experiments. **(C)** MTT assay determining the IC50 of Ctrl-C2C12 and P3F-C2C12 cells treated with ascending concentrations of H_2_O_2_ for 24 h. Data are reported as mean of three independent experiments ± SD. Asterisks denote a statistically significant difference (*p*-value < 0.05); (*) compared to control Exo-free treated cells.

Of the 26 enriched proteins in P3F-C2C12-derived exosomes, we found that 4 proteins are implicated in the ROS pathway. These proteins include prostaglandin reductase 1 (PtgR1), which has an oxidoreductase activity that can reduce cytotoxic unsaturated aldehydes and ketones produced by lipid peroxidation during oxidative stress (38); glutathione S-transferase (GST), which detoxifies secondary metabolites produced by interaction between ROS and cellular components (39); superoxide dismutase (SOD1), which is an antioxidant enzyme that reduces superoxide (O${}_{2}^{-}$) and prevents its accumulation (40); and malate dehydrogenase, which is activated by ROS and seems to act as a redox regulator in response to environmental changes (41). These 4 enzymes mainly act by decreasing ROS levels. Since ROS contributes to oxidative stress, we hypothesized that the P3F fusion protein may be influencing redox homeostasis and response to oxidative stress. Indeed, the expression levels of the 3 main subunits of the NADPH oxidase complex, which is a main enzymatic producer of ROS: gp91, gp47, and Nox1 were decreased in P3F-transduced C2C12 cells compared to control cells (Figure 5A). Several bands appear for Nox1 protein due to different glycosylation sites (42). This result was confirmed by flow cytometry analysis using the DCF-DA dye to detect ROS levels, where we found that P3F-C2C12 cells have markedly decreased proportion of DCF-positive cells as compared to Ctrl-C2C12 cells, demonstrating lower ROS levels in response to P3F expression (Figure 5B).

Next, we subjected the transduced cells to oxidative stress in order to test the protective effect of the fusion protein. After treating the cells with different concentrations of H_2_O_2_ (0 to 500 μM) for 24 h, P3F-C2C12 cells showed a higher IC_50_ (68 μM) than Ctrl-C2C12 cells (36.8 μM; Figure 5C). Thus, P3F-C2C12 cells are more tolerant to endogenous and exogenous oxidative stress than control cells. These results show that the PAX3-FOXO1 fusion protein can decrease ROS levels in host cells, likely contributing to cell survival.

**FIGURE 6:**
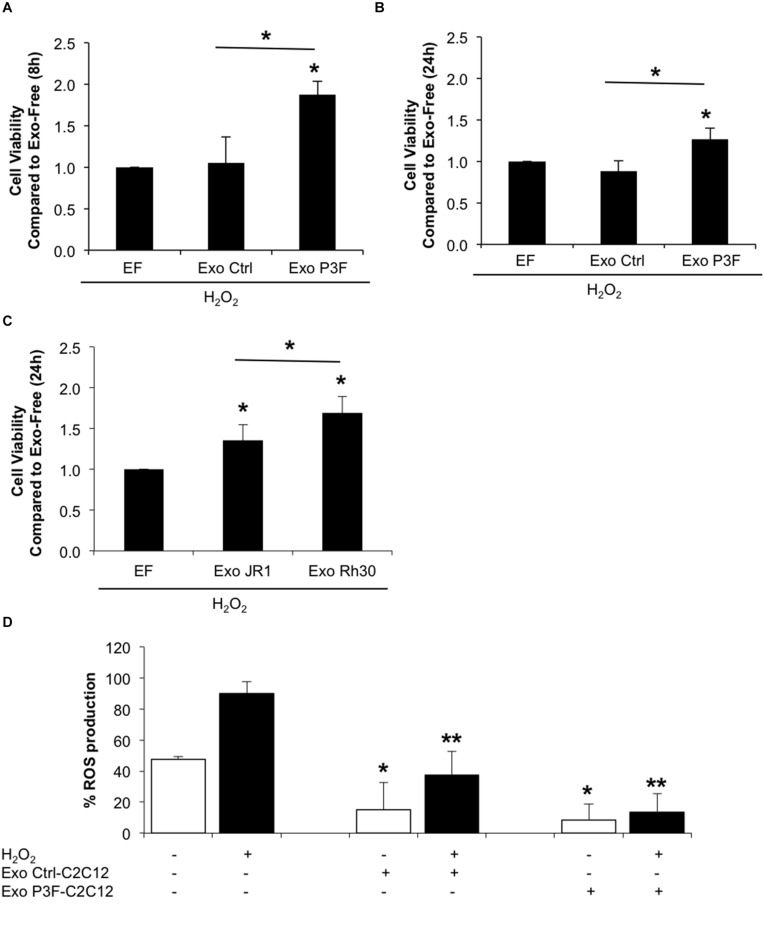
P3F confers protection from oxidative stress in both autocrine and paracrine manner through exosomes. **(A,B)** Histograms representing the ratio of living cells in each condition relative to the exo-free control, after 8 h **(A)** and 24 h **(B)** of H_2_O_2_ treatment assessed by Trypan blue exclusion assay. **(C)** Histogram representing the ratio of living cells in each condition relative to the exo-free control, after 24 h of H_2_O_2_ treatment assessed by Trypan blue exclusion assay. Data are reported as mean of three independent experiments ± SD. Asterisks denote a statistically significant difference (*p*-value < 0.05). **(D)** Histogram displaying the percentage of DCF positive cells in each condition. Data are reported as mean of three independent experiments ± SD. Asterisks denote a statistically significant difference (*p*-value < 0.05); (*) compared to control Exo-free treated cells and (**) compared to H_2_O_2_ treated cells.

Since P3F-C2C12 cells were found to be tolerant to H_2_O_2_-induced oxidative stress, we next investigated whether this resistance is provided to recipient cells through exosome cargo. Treating C2C12 myoblasts with a combination of H_2_O_2_ and P3F-C2C12-derived exosomes at 10X concentration showed significantly higher viability in comparison to cells treated with H_2_O_2_ and Ctrl-C2C12-derived exosomes (1.88 *versus* 1.05 at 8 h; and 1.27 *versus* 0.88 at 24 h**;** Figures 6A,B). Interestingly, this protective effect was also observed when treating human ERMS JR1 cells with a combination of H_2_O_2_ and ARMS Rh30-derived exosomes showing a significantly higher viability when compared to cells treated with H_2_O_2_ and JR1-derived exosomes (Figure 6C). This confirms a protection conferred by FPRMS-derived exosomes to recipient cells in conditions of oxidative stress.

To clarify whether this protection was related to decreased levels of ROS in cells treated with P3F-modulated exosomes as opposed to control exosomes, we pretreated C2C12 cells with exosomes for 48 h, and then exposed them to H_2_O_2_, and evaluated ROS levels by DCF-DA. Treatment with exosomes, whether control or PAX3-FOXO1-modulated, both had an inhibitory effect on ROS production (Figure 6D). The induction of ROS observed by treating the recipient cells with H_2_O_2_ was significantly decreased when co-treated with either exosome type, but to a much higher extent when treated with P3F-C2C12-derived exosomes. Indeed, P3F-C2C12-derived exosomes were able to decrease ROS levels by 6.6 fold whereas Ctrl-C2C12-derived exosomes decreased it only by 2.4 fold when compared to H_2_O_2_ treatment alone (Figure 6D). These observations define a clear role of P3F-modulated exosomes in protecting recipient cells, even in the presence of oxidative stress, from elevated intracellular ROS levels.

## Discussion

Rhabdomyosarcoma is an aggressive pediatric cancer with limited improvement in overall survival outcomes in patients with aggressive subtypes and advanced stages (1). Presence of the P3F or the less common PAX7-FOXO1 fusion oncoprotein is associated with increased incidence of metastasis and disease aggressiveness (43, 44). There is therefore a pressing need for identifying effective therapeutic targets and biomarkers for these fusion gene positive (FP) RMS.

Exosomes can act *via* paracrine signaling on neighboring stromal cells altering their behavior and enhancing tumor growth (9, 15, 45). Both FPRMS and FNRMS cells secrete quantifiable amounts of exosomes with specific cargo that can influence cancer-related processes including cell motility and angiogenesis (6), and fusion status plays a role in modulating exosome cargo to favor tumor invasion (6, 12, 18).

In this study, we investigated the effects of P3F on myoblasts using C2C12 cells, and studied its role in modulation of exosome protein content and paracrine signaling. Both the previously identified deregulated miRNA revealed by microarray profiling (18) and the deregulated exosomal proteins identified in this study were associated with pathways important in cell plasticity including cellular motility, cell survival, and protection from oxidative stress. The networks identified centered on insulin, FSH and TP53, all of which have previously been associated with the expression of P3F or implicated in RMS tumor progression. For instance, both insulin, and insulin-like growth factors (IGF) regulate skeletal muscle differentiation, and their pathways are deregulated in RMS (46, 47). Using C2C12 myoblasts, Wang et al. showed that P3F and IGF2 cooperate to induce cell proliferation and invasion (48). Additionally, P3F has been shown to up-regulate the expression of IGF1 receptor and IGF2, both contributing to FPRMS proliferation (49). P3F also induces aberrant and elevated expression of GLUT4, enhancing cellular response to insulin (50). As for FSH, its receptors are expressed in both FPRMS and FNRMS human cell lines, as well as in primary tumor samples. Stimulation of RMS cells with FSH results in enhanced proliferation and chemotaxis, indicating a role in promoting RMS progression (51). FSH down-regulates FOXO1 mRNA levels, and FSH receptor activation results in FOXO1 phosphorylation, which inhibits FOXO1-mediated repression of apoptosis and promotes cell survival (52, 53). However, whether P3F contributes to FSH-mediated repression of wild type FOXO1 is yet to be determined. Finally, p53 pathway deregulation has been observed in RMS and contributes to tumor progression and relapse (54–56). While p53 pathway deregulation in FNRMS is mainly via inactivation mutations in *TP53*, in FPRMS its deregulation is likely mediated by the fusion oncoprotein, as PAX proteins have been shown to transcriptionally inhibit p53 (57). Moreover, *TP53* loss markedly increases the progression of P3F-driven tumors in mice (58). Indeed, 85% of human tumors harboring P3F fusion were associated with *p53* inactivation, suggesting a cooperativity between the fusion protein and p53 pathway deregulation in driving tumorigenesis (59). In concordance, our results further reveal that P3F expression leads to exosomal-mediated deregulation of these signaling pathways, likely contributing to their deregulation in recipient cells.

Functionally, exosomes of P3F-transduced cells modulated recipient cell plasticity to favor proliferation, invasion, angiogenesis and stemness, as well as protect them from the hostile conditions of the tumor microenvironment (TME). Angiogenesis and neovascularization are indicative of cell plasticity that allows tumor growth and cell survival in hypoxic conditions of the TME (19, 60). Ploeger et al. showed that fibroblasts exhibit a high dynamic plasticity in wound healing resulting from a combination of recruitment and cell proliferation, a plasticity that seems to be regulated by the micro-environment (61). P3F-modulated exosomes were able to induce tube formation and migration of recipient HUVECs, as well as induce degradation of matrix via activation of MMPs, indicating a role for these exosomes in inducing angiogenesis and alteration of the stromal matrix.

Escaping cell death is another indication of cellular plasticity in response to change in physiological conditions (21, 62). Pathway analysis by IPA of the proteins and miRNA modulated in exosomes by P3F showed a common impact on reducing apoptosis and increasing cell viability (see Figure 3A: 10 out of 25 pathways are implicated in death pathways). This is further evidenced by the observed resistance of cells treated with P3F-modulated exosomes to apoptosis in response to exogenous oxidative stress, which may be impacted by both modulation of oxidative stress pathway, as well as resistance to apoptosis in general. Of note, elevated ROS levels are detected in almost all cancers, due at least in part to the high metabolic activity and mitochondrial dysfunction of cancer cells. In comparison, normal cells exhibit moderate levels of ROS, enough for them to act as second messengers in signal transduction, and contribute to physiological processes within these cells (63). To cope with the ever-present state of oxidative stress, cancer cells up-regulate their antioxidant pathways to escape cell death (64). For instance, in RMS, elevated ROS levels are compensated by an up-regulation of ROS-scavenging pathways, thus conferring resistance to ROS-induced stress (65). Moreover, up-regulation of ROS production in RMS cells following radiation therapy was rapidly compensated by an up-regulation of nuclear factor erythroid 2-related factor (NRF2) levels which, in turn, promotes the expression of antioxidant enzymes and miRNA that protected RMS cells from ROS-induced DNA damage (66). This has been suggested as a possible Achilles’ heel of cancer cells, as they may be susceptible to treatments that can alter their redox homeostasis, either by further increasing intracellular ROS levels or by targeting antioxidant abilities (64, 67). RMS might be particularly vulnerable to this pathway, as demonstrated by Chen et al. using a high-throughput screen. They reasoned that this sensitivity may be especially applicable to RMS, as it derives from a skeletal muscle cell lineage, since skeletal muscle cells have unique metabolic properties including a high aerobic activity and a robust antioxidant defense system against excessive ROS, which make them particularly sensitive to redox-altering agents (55). In fact, analysis of RMS samples revealed oxidative stress-induced genomic mutations that enhance RMS cell survival against oxidative damage whereas combinational targeting of the major antioxidative pathways up-regulated in RMS, the thioredoxin (TRX) and glutathione (GSH) synthesis pathways, using GSH-depleting agents and TRX-reductase inhibitors seems to induce cancer cell death (55, 64). Therapeutic interventions using agents that can increase ROS production also offer promising therapeutic results. For instance, carfilzomib and alvocidib, in addition to synthetic statins, where shown to act in synergism with HDAC inhibitors by increasing oxidative stress showing effective activity against ERMS xenografts (55). Erastin, another GSH-depleting agent that subsequently up-regulates ROS production, also induces RMS cell death (67). Notably, following ROS-inducing radiation therapy, FPRMS cells were found to produce higher levels of antioxidant miRNA including *miR-22*, *miR-210*, and *miR-375* compared to FNRMS, where the expression of *miR-375* was only observed in FPRMS, and certain antioxidant enzymes including catalase and glutathione peroxidase 4 (66). Moreover, Martin et al. demonstrated that FPRMS treatment with fenretinide induces ROS production accompanied by a reduction in P3F transcriptional activity. ROS-induction in FPRMS using Triterpenoid down-regulated the expression of transcription factors including P3F suggesting the need to determine the link between oxidative stress and P3F in RMS (68, 69). Altogether, RMS cells have been shown to modulate intracellular signaling pathways to maintain redox homeostasis, an aspect of cellular plasticity that protects against oxidative damage and highlights the importance of using redox-based therapeutic strategies in combination with conventional chemotherapeutic treatments against RMS. Our results further underline this finding, and specifically implicate the P3F protein in modulating oxidative stress response, not only in an autocrine manner in RMS cells, but also through paracrine effects on stromal cells *via* exosomes. We observed lower ROS levels within the P3F transduced C2C12 cells, with enhanced survival upon treatment with H_2_O_2_. We identified an enrichment of 4 REDOX proteins in exosomes in response to P3F that may be contributing to the observed decrease in intracellular ROS levels in recipient cells, confirming paracrine protective effects of the fusion oncoprotein via exosomes. Indeed, previous studies have implicated exosomes in modulating response to oxidative stress and conferring antioxidant resistance in several settings (27, 70), and our results now demonstrate the additional effect of oncoproteins such as P3F in further augmenting this response.

Altogether, our results indicate that the P3F oncoprotein down-regulates ROS levels, exerting a protective effect against oxidative stress-induced cell death in an autocrine manner. This effect can also be transferred to stromal and other cells through secreted exosomes that also affect recipient cell invasion, migration, and pro-angiogenic properties. We have shown that the P3F fusion protein is able to modulate the content of exosomes secreted by fusion-positive cells and that these exosomes seem to confer cellular plasticity and invasive properties, including augmentation of pro-angiogenic signaling. Detailed understanding of the pathways through which P3F alters the metabolic response of cells may uncover novel therapeutic targets for enhancing cell death in response to metabolic stress in this setting, and should be the focus of future studies.

## Data Availability Statement

The original contributions presented in the study are publicly available. This data can be found here: ProteomeXchange Consortium via the PRIDE partner repository (Accession Number: PXD017543).

## Author Contributions

AF, FG, AA, and FA conducted most of the experiments and data analysis. AF, FR, and FG helped in drafting the manuscript. FK, YM, JZ, and RZ performed and analyzed the LC-MS/MS experiments. NH helped in the design of the flow cytometry experiments and their analysis. SG and RS oversaw the design and coordination of the studies, and drafted the manuscript. All authors read and approved the final manuscript.

## Conflict of Interest

The authors declare that the research was conducted in the absence of any commercial or financial relationships that could be construed as a potential conflict of interest.

## Footnotes

1 http://pantherdb.org

## References

[1] Breneman JC Lyden E Pappo AS Link MP Anderson JR Parham DM Prognostic factors and clinical outcomes in children and adolescents with metastatic rhabdomyosarcoma-a report from the intergroup rhabdomyosarcoma study IV. *J Clin Oncol.* (2003) 21:78–84. 10.1200/JCO.2003.06.129 12506174

[2] Rudzinski ER Anderson JR Hawkins DS Skapek SX Parham DM Teot LA. The world health organization classification of skeletal muscle tumors in pediatric rhabdomyosarcoma a report from the children’s oncology group. *Arch Pathol Lab Med.* (2015) 139:1281–7. 10.5858/arpa.2014-0475-OA 25989287 PMC4651658

[3] Williamson D Missiaglia E De Reyniès A Pierron G Thuille B Palenzuela G Fusion gene-negative alveolar rhabdomyosarcoma is clinically and molecularly indistinguishable from embryonal rhabdomyosarcoma. *J Clin Oncol.* (2010) 28:2151–8. 10.1200/JCO.2009.26.3814 20351326

[4] Rudzinski ER Anderson JR Chi YY Gastier-Foster JM Astbury C Barr FG Histology, fusion status, and outcome in metastatic rhabdomyosarcoma: a report from the children’s oncology group. *Pediatr Blood Cancer.* (2017) 64:e26645. 10.1002/pbc.26645 28521080 PMC5647228

[5] Shern JF Chen L Chmielecki J Wei JS Patidar R Rosenberg M Comprehensive genomic analysis of rhabdomyosarcoma reveals a landscape of alterations affecting a common genetic axis in fusion-positive and fusion-negative tumors. *Cancer Discov.* (2014) 4:216–31. 10.1158/2159-8290.CD-13-0639 24436047 PMC4462130

[6] Ghayad SE Rammal G Ghamloush F Basma H Nasr R Diab-Assaf M Exosomes derived from embryonal and alveolar rhabdomyosarcoma carry differential miRNA cargo and promote invasion of recipient fibroblasts. *Sci Rep.* (2016) 6:37088. 10.1038/srep37088 27853183 PMC5112573

[7] Diomedi-Camassei F Boldrini R Ravà L Donfrancesco A Boglino C Messina E Different pattern of matrix metalloproteinases expression in alveolar versus embryonal rhabdomyosarcoma. *J Pediatr Surg.* (2004) 39:1673–9. 10.1016/j.jpedsurg.2004.07.014 15547833

[8] Théry C Zitvogel L Amigorena S. Exosomes: composition, biogenesis and function. *Nat Rev Immunol.* (2002) 2:569–79. 10.1038/nri855 12154376

[9] Miller IV Raposo G Welsch U Prazeres da Costa O Thiel U Lebar M First identification of Ewing’s sarcoma-derived extracellular vesicles and exploration of their biological and potential diagnostic implications. *Biol Cell.* (2013) 105:289–303. 10.1111/boc.201200086 23521563

[10] Raimondi L De Luca A Gallo A Costa V Russelli G Cuscino N Osteosarcoma cell-derived exosomes affect tumor microenvironment by specific packaging of microRNAs. *Carcinogenesis.* (2019) 41:666–77. 10.1093/carcin/bgz130 31294446

[11] Peinado H Alečković M Lavotshkin S Matei I Costa-Silva B Moreno-Bueno G Melanoma exosomes educate bone marrow progenitor cells toward a pro-metastatic phenotype through MET. *Nat Med.* (2012) 18:883–91. 10.1038/nm.2753 22635005 PMC3645291

[12] Rammal G Fahs A Kobeissy F Mechref Y Zhao J Zhu R Proteomic profiling of rhabdomyosarcoma-derived exosomes yield insights into their functional role in paracrine signaling. *J Proteome Res.* (2019) 18:3567–79. 10.1021/acs.jproteome.9b00157 31448612

[13] Hoshino A Costa-Silva B Shen TL Rodrigues G Hashimoto A Tesic Mark M Tumour exosome integrins determine organotropic metastasis. *Nature.* (2015) 527:329–35. 10.1038/nature15756 26524530 PMC4788391

[14] Zhang Y Wang XF. A niche role for cancer exosomes in metastasis. *Nat Cell Biol.* (2015) 17:709–11. 10.1038/ncb3181 26022917

[15] Lobb RJ Lima LG Möller A. Exosomes: key mediators of metastasis and pre-metastatic niche formation. *Semin Cell Dev Biol.* (2017) 67:3–10. 10.1016/j.semcdb.2017.01.004 28077297

[16] Paolillo M Schinelli S. Integrins and exosomes, a dangerous liaison in cancer progression. *Cancers.* (2017) 9:95. 10.3390/cancers9080095 28933725 PMC5575598

[17] Maji S Chaudhary P Akopova I Nguyen PM Hare RJ Gryczynski I Exosomal annexin II promotes angiogenesis and breast cancer metastasis. *Mol Cancer Res.* (2017) 15:93–105. 10.1158/1541-7786.MCR-16-0163 27760843 PMC5215956

[18] Ghamloush F Ghayad SE Rammal G Fahs A Ayoub AJ Merabi Z The PAX3-FOXO1 oncogene alters exosome miRNA content and leads to paracrine effects mediated by exosomal miR-486. *Sci Rep.* (2019) 9:14242. 10.1038/s41598-019-50592-4 31578374 PMC6775163

[19] Yuan S Norgard RJ Stanger BZ. Cellular plasticity in cancer. *Cancer Discov.* (2019) 9:837–51. 10.1158/2159-8290.cd-19-0015 30992279 PMC6606363

[20] Pardali E Van Der Schaft DWJ Wiercinska E Gorter A Hogendoorn PCW Griffioen AW Critical role of endoglin in tumor cell plasticity of Ewing sarcoma and melanoma. *Oncogene.* (2011) 30:334–45. 10.1038/onc.2010.418 20856203

[21] da Silva-Diz V Lorenzo-Sanz L Bernat-Peguera A Lopez-Cerda M Muñoz P. Cancer cell plasticity: impact on tumor progression and therapy response. *Semin Cancer Biol.* (2018) 53:48–58. 10.1016/j.semcancer.2018.08.009 30130663

[22] Nakahata K Uehara S Nishikawa S Kawatsu M Zenitani M Oue T Aldehyde dehydrogenase 1 (ALDH1) is a potential marker for cancer stem cells in embryonal rhabdomyosarcoma. *PLoS One.* (2015) 10:e0125454. 10.1371/journal.pone.0125454 25915760 PMC4411144

[23] Walter D Satheesha S Albrecht P Bornhauser BC D’Alessandro V Oesch SM CD133 positive embryonal rhabdomyosarcoma stem-like cell population is enriched in rhabdospheres. *PLoS One.* (2011) 6:e19506. 10.1371/journal.pone.0019506 21602936 PMC3094354

[24] Sosa V Moliné T Somoza R Paciucci R Kondoh H LLeonart ME. Oxidative stress and cancer: an overview. *Ageing Res Rev.* (2013) 12:376–90. 10.1016/j.arr.2012.10.004 23123177

[25] Nisimoto Y Diebold BA Constentino-Gomes D Lambeth JD. Nox4: a hydrogen peroxide-generating oxygen sensor. *Biochemistry.* (2014) 53:5111–20. 10.1021/bi500331y 25062272 PMC4131900

[26] Kumari S Badana AK Murali Mohan G Shailender G Malla RR. Reactive oxygen species: a key constituent in cancer survival. *Biomark Insights.* (2018) 13:1177271918755391. 10.1177/1177271918755391 29449774 PMC5808965

[27] Eldh M Ekström K Valadi H Sjöstrand M Olsson B Jernås M Exosomes communicate protective messages during oxidative stress; possible role of exosomal shuttle RNA. *PLoS One.* (2010) 5:e15353. 10.1371/journal.pone.0015353 21179422 PMC3003701

[28] Chettimada S Lorenz DR Misra V Dillon ST Reeves RK Manickam C Exosome markers associated with immune activation and oxidative stress in HIV patients on antiretroviral therapy. *Sci Rep.* (2018) 8:7227. 10.1038/s41598-018-25515-4 29740045 PMC5940833

[29] Hinson ARP Jones R Lisa LE Belyea BC Barr FG Linardic CM. Human rhabdomyosarcoma cell lines for rhabdomyosarcoma research: utility and pitfalls. *Front Oncol.* (2013) 3:183. 10.3389/fonc.2013.00183 23882450 PMC3713458

[30] Bonnet A Lagarrigue S Liaubet L Robert-Granié C SanCristobal M Tosser-Klopp G. Pathway results from the chicken data set using GOTM, pathway studio and ingenuity softwares. *BMC Proc.* (2009) 3 (Suppl. 4):S11. 10.1186/1753-6561-3-s4-s11 19615111 PMC2712741

[31] Yuryev A Kotelnikova E Daraselia N. Ariadne’s chemeffect and pathway studio knowledge base. *Expert Opin Drug Discov.* (2009) 4:1307–18. 10.1517/17460440903413488 23480468

[32] Toth M Sohail A Fridman R. Assessment of gelatinases (MMP-2 and MMP-9) by gelatin zymography. *Methods Mol Biol.* (2012) 878:121–35. 10.1007/978-1-61779-854-2_8 22674130

[33] Kitajima S Kohno S Kondoh A Sasaki N Nishimoto Y Li F Undifferentiated state induced by Rb-p53 double inactivation in mouse thyroid neuroendocrine cells and embryonic fibroblasts. *Stem Cells.* (2015) 33:1657–69. 10.1002/stem.1971 25694388

[34] Marshall AD Lagutina I Grosveld GC. PAX3-FOXO1 induces cannabinoid receptor 1 to enhance cell invasion and metastasis. *Cancer Res.* (2011) 71:7471–80. 10.1158/0008-5472.CAN-11-0924 22037868

[35] Deschênes-Simard X Kottakis F Meloche S Ferbeyre G. ERKs in cancer: friends or foes? *Cancer Res.* (2014) 74:412–9. 10.1158/0008-5472.CAN-13-2381 24408923

[36] Ma XM Blenis J. Molecular mechanisms of mTOR-mediated translational control. *Nat Rev Mol Cell Biol.* (2009) 10:307–18. 10.1038/nrm2672 19339977

[37] Hu Y Yan C Mu L Huang K Li X Tao D Fibroblast-derived exosomes contribute to chemoresistance through priming cancer stem cells in colorectal cancer. *PLoS One.* (2015) 10:e0125625. 10.1371/journal.pone.0125625 25938772 PMC4418721

[38] Dick RA Kwak MK Sutter TR Kensler TW. Antioxidative function and substrate specificity of NAD(P)H-dependent alkenal/one oxidoreductase. A new role for leukotriene B4 12-hydroxydehydrogenase/15-oxoprostaglandin 13-reductase. *J Biol Chem.* (2001) 276:40803–10. 10.1074/jbc.M105487200 11524419

[39] Hayes JD Pulford DJ. The glut athione s-transferase supergene family: regulation of GST and the contribution of the lsoenzymes to cancer chemoprotection and drug resistance part II. *Crit Rev Biochem Mol Biol.* (1995) 30:445–600. 10.3109/10409239509083492 8770536

[40] Fukai T Ushio-Fukai M. Superoxide dismutases: role in redox signaling, vascular function, and diseases. *Antioxidants Redox Signal.* (2011) 15:1583–606. 10.1089/ars.2011.3999 21473702 PMC3151424

[41] Scheibe R Backhausen JE Emmerlich V Holtgrefe S. Strategies to maintain redox homeostasis during photosynthesis under changing conditions. *J Exp Bot.* (2005) 56:1481–9. 10.1093/jxb/eri181 15851411

[42] Matsumoto M Katsuyama M Iwata K Ibi M Zhang J Zhu K Characterization of N-glycosylation sites on the extracellular domain of NOX1/NADPH oxidase. *Free Radic Biol Med.* (2014) 68:196–204. 10.1016/j.freeradbiomed.2013.12.013 24361341

[43] Parham DM Barr FG. Classification of rhabdomyosarcoma and its molecular basis. *Adv Anat Pathol.* (2013) 20:387–97. 10.1097/PAP.0b013e3182a92d0d 24113309 PMC6637949

[44] Hawkins DS Gupta AA Rudzinski ER. What is new in the biology and treatment of pediatric rhabdomyosarcoma? *Curr Opin Pediatr.* (2014) 26:50–6. 10.1097/MOP.0000000000000041 24326270 PMC4096484

[45] Tai YL Chen KC Hsieh JT Shen TL. Exosomes in cancer development and clinical applications. *Cancer Sci.* (2018) 109:2364–74. 10.1111/cas.13697 29908100 PMC6113508

[46] Blandford MC Barr FG Lynch JC Randall RL Qualman SJ Keller C. Rhabdomyosarcomas utilize developmental, myogenic growth factors for disease advantage: a report from the children’s oncology group. *Pediatr Blood Cancer.* (2006) 46:329–38. 10.1002/pbc.20466 16261596

[47] Tarnowski M Tkacz M Zgutka K Bujak J Kopytko P Pawlik A. Picropodophyllin (PPP) is a potent rhabdomyosarcoma growth inhibitor both in vitro and in vivo. *BMC Cancer.* (2017) 17:532. 10.1186/s12885-017-3495-y 28793874 PMC5550998

[48] Wang W Kumar P Wang W Epstein J Helman L Moore JV Insulin-like growth factor II and PAX3-FKHR cooperate in the oncogenesis of rhabdomyosarcoma. *Cancer Res.* (1998) 58:4426–33. 9766674

[49] Ayalon D Glaser T Werner H. Transcriptional regulation of IGF-I receptor gene expression by the PAX3-FKHR oncoprotein. *Growth Horm IGF Res.* (2001) 11:289–97. 10.1054/ghir.2001.0244 11735247

[50] Armoni M Quon MJ Maor G Avigad S Shapiro DN Harel C PAX3/forkhead homolog in rhabdomyosarcoma oncoprotein activates glucose transporter 4 gene expression in vivo and in vitro. *J Clin Endocrinol Metab.* (2002) 87:5312–24. 10.1210/jc.2002-020318 12414908

[51] Poniewierska-Baran A Schneider G Sun W Abdelbaset-Ismail A Barr FG Ratajczak MZ. Human rhabdomyosarcoma cells express functional pituitary and gonadal sex hormone receptors: therapeutic implications. *Int J Oncol.* (2016) 48:1815–24. 10.3892/ijo.2016.3439 26983595 PMC4809652

[52] Nechamen CA Thomas RM Cohen BD Acevedo G Poulikakos PI Testa JR Human follicle-stimulating hormone (FSH) receptor interacts with the adaptor protein APPL1 in HEK 293 Cells: potential involvement of the PI3K pathway in FSH signaling1. *Biol Reprod.* (2004) 71:629–36. 10.1095/biolreprod.103.025833 15070827

[53] Rosairo D Kuyznierewicz I Findlay J Drummond A. Transforming growth factor-β: its role in ovarian follicle development. *Reproduction.* (2008) 136:799–809. 10.1530/REP-08-0310 18780765

[54] Taylor AC Shu L Danks MK Poquette CA Shetty S Thayer MJ p53 mutation and MDM2 amplification frequency in pediatric rhabdomyosarcoma tumors and cell lines. *Med Pediatr Oncol.* (2000) 35:96–103. 10.1002/1096-911X(200008)35:23.0.CO;2-Z 10918230

[55] Chen X Stewart E Shelat AA Qu C Bahrami A Hatley M Targeting oxidative stress in embryonal rhabdomyosarcoma. *Cancer Cell.* (2013) 24:710–24. 10.1016/j.ccr.2013.11.002 24332040 PMC3904731

[56] Mai PL Best AF Peters JA DeCastro RM Khincha PP Loud JT Risks of first and subsequent cancers among TP53 mutation carriers in the National Cancer Institute Li-Fraumeni syndrome cohort. *Cancer.* (2016) 122:3673–81. 10.1002/cncr.30248 27496084 PMC5115949

[57] Stuart ET Haffner R Oren M Gruss P. Loss of p53 function through PAX-mediated transcriptional repression. *EMBO J.* (1995) 14:5638–45. 10.1002/j.1460-2075.1995.tb00251.x 8521821 PMC394679

[58] Keller C Arenkiel BR Coffin CM El-Bardeesy N DePinho RA Capecchi MR. Alveolar rhabdomyosarcomas in conditional Pax3:Fkhr mice: cooperativity of Ink4a/ARF and Trp53 loss of function. *Genes Dev.* (2004) 18:2614–26. 10.1101/gad.1244004 15489287 PMC525542

[59] Abraham J Nuñez-Álvarez Y Hettmer S Carrió E Chen HIH Nishijo K Lineage of origin in rhabdomyosarcoma informs pharmacological response. *Genes Dev.* (2014) 28:1578–91. 10.1101/gad.238733.114 25030697 PMC4102765

[60] Mihic-Probst D Ikenberg K Tinguely M Schraml P Behnke S Seifert B Tumor cell plasticity and angiogenesis in human melanomas. *PLoS One.* (2012) 7:e33571. 10.1371/journal.pone.0033571 22442699 PMC3307737

[61] Ploeger DT Hosper NA Schipper M Koerts JA De Rond S Bank RA. Cell plasticity in wound healing: paracrine factors of M1/M2 polarized macrophages influence the phenotypical state of dermal fibroblasts. *Cell Commun Signal.* (2013) 11:29. 10.1186/1478-811X-11-29 23601247 PMC3698164

[62] Taddei ML Giannoni E Comito G Chiarugi P. Microenvironment and tumor cell plasticity: an easy way out. *Cancer Lett.* (2013) 341:80–96. 10.1016/j.canlet.2013.01.042 23376253

[63] Liou GY Storz P. Reactive oxygen species in cancer. *Free Radic Res.* (2010) 44:479–96. 10.3109/10715761003667554 20370557 PMC3880197

[64] Habermann KJ Grünewald L van Wijk S Fulda S. Targeting redox homeostasis in rhabdomyosarcoma cells: GSH-depleting agents enhance auranofin-induced cell death. *Cell Death Dis.* (2017) 8:e3067. 10.1038/cddis.2017.412 28981107 PMC5680568

[65] Fan TWM Kucia M Jankowski K Higashi RM Ratajczak J Ratajczak MZ Rhabdomyosarcoma cells show an energy producing anabolic metabolic phenotype compared with primary myocytes. *Mol Cancer.* (2008) 7:79. 10.1186/1476-4598-7-79 18939998 PMC2577687

[66] Marampon F Codenotti S Megiorni F Del Fattore A Camero S Gravina GL NRF2 orchestrates the redox regulation induced by radiation therapy, sustaining embryonal and alveolar rhabdomyosarcoma cells radioresistance. *J Cancer Res Clin Oncol.* (2019) 145:881–93. 10.1007/s00432-019-02851-0 30701326 PMC11810283

[67] Dächert J Ehrenfeld V Habermann K Dolgikh N Fulda S. Targeting ferroptosis in rhabdomyosarcoma cells. *Int J Cancer.* (2020) 146:510–20. 10.1002/ijc.32496 31173656

[68] Herrero Martín D Boro A Schäfer BW. Cell-based small-molecule compound screen identifies fenretinide as potential therapeutic for translocation-positive rhabdomyosarcoma. *PLoS One.* (2013) 8:e55072. 10.1371/journal.pone.0055072 23372815 PMC3555977

[69] Kasiappan R Jutooru I Mohankumar K Karki K Lacey A Safe S. Reactive oxygen species (ROS)-inducing triterpenoid inhibits rhabdomyosarcoma cell and tumor growth through targeting SP transcription factors. *Mol Cancer Res.* (2019) 17:794–805. 10.1158/1541-7786.MCR-18-1071 30610105 PMC6397684

[70] Saeed-Zidane M Linden L Salilew-Wondim D Held E Neuhoff C Tholen E Cellular and exosome mediated molecular defense mechanism in bovine granulosa cells exposed to oxidative stress. *PLoS One.* (2017) 12:e0187569. 10.1371/journal.pone.0187569 29117219 PMC5678720

