# Supplementary material for: Effects of the Oncoprotein PAX3-FOXO1 on Modulation of Exosomes Function and Protein Content: Implications on Oxidative Stress Protection and Enhanced Plasticity
Source: Front Oncol. 2020 Oct 1;10:1784. doi: 10.3389/fonc.2020.01784 (PMC7560303; doi:10.3389/fonc.2020.01784)
Supplement: Supplementary file 1 [file Image_1.pdf]

frontiers

Effects of the oncoprotein PAX3-FOXO1 on modulation of exosomes function and protein content: Implications on oxidative stress protection and enhanced plasticity.

Assil Fahs[1,2], Farah Ramadan[1], Farah Ghamloush[3], Abeer J. Ayoub[1,2], Fatima Ali Ahmad[1,2], Firas Kobeissy[4], Yehia Mechref [5], Jingfu Zhao[5], Rui Zhu[5], Nader Hussein[6], Raya Saab[2,3]*, Sandra E. Ghayad[1]*.

[1]Department of Biology, Faculty of Science II, Lebanese University, Fanar, Lebanon.

[2]Department of Anatomy, Cell Biology and Physiology, American University of Beirut, Beirut, Lebanon.

[3]Department of Pediatrics and Adolescent Medicine, Children's Cancer Institute, American University of Beirut, Beirut, Lebanon.

[4]Department of Biochemistry & Molecular Genetics, Faculty of Medicine, American University of Beirut, Lebanon.

[5]Department of Chemistry & Biochemistry, Texas Tech University, Lubbock, United States.

[6]Lebanese University, Faculty of Sciences, Cancer biology Stem Cells and Molecular Immunology Laboratory, Hadath, Beirut, Lebanon.

***Correspondence:**

Sandra E. Ghayad, Lebanese University, Faculty of Science II, Department of Biology, Fanar, Jdeidet, P.O. Box 90656, Lebanon. Phone: +961-1-686981. Fax: +961-1-377384. Email: sandra.ghayad@ul.edu.lb.

Raya Saab, MD, Department of Pediatrics and Adolescent Medicine, Children's Cancer Institute, American University of Beirut Medical Center, Riad El Solh Street, Beirut 1107 2020, Lebanon. Tel.: +961-1-350000. Email: rs88@aub.edu.lb.

Keywords: Rhabdomyosarcoma, PAX3-FOXO1, exosomes, oxidative stress, plasticity.

Supplementary Material

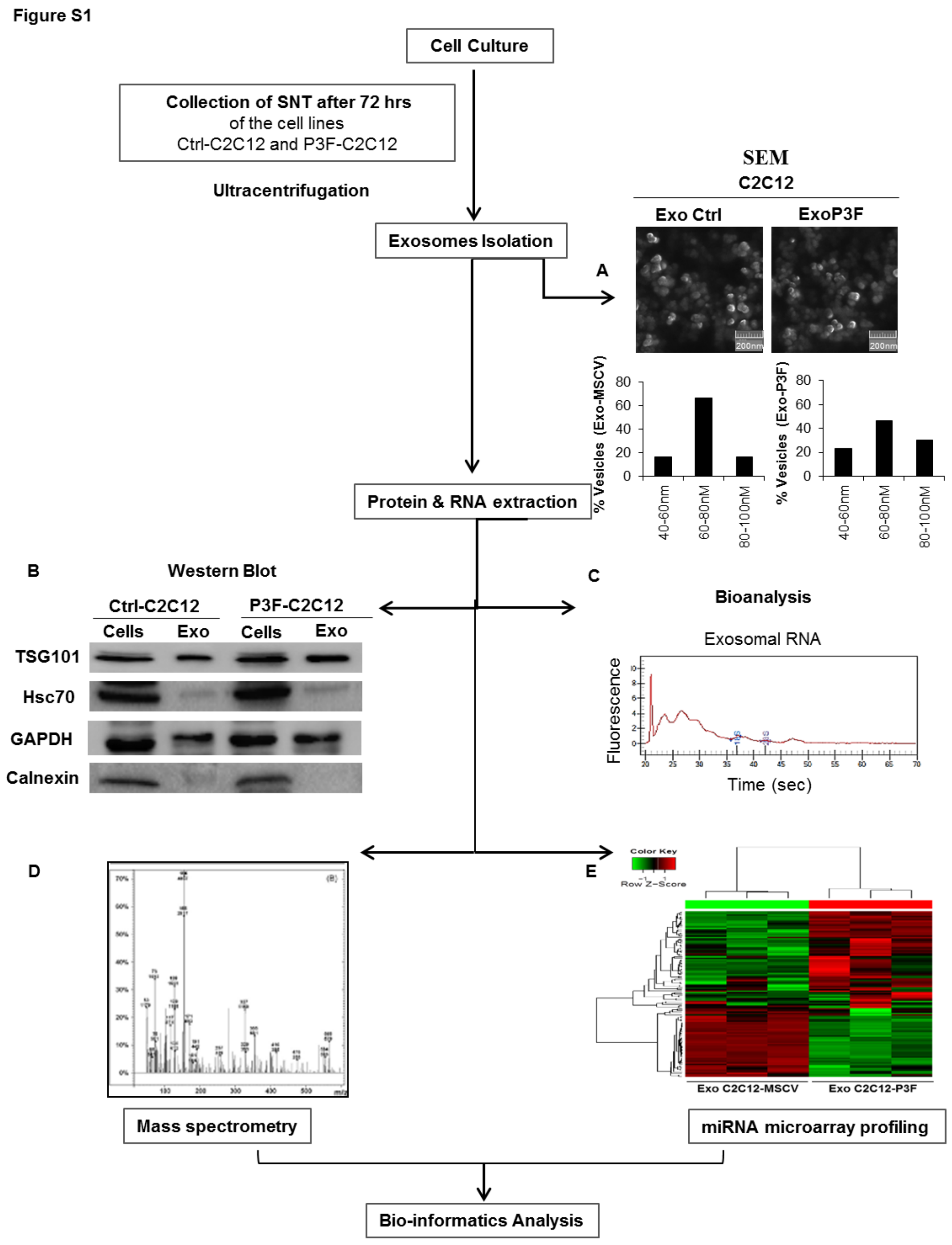

Supplementary Figure 1: Isolation and characterization of C2C12-derived exosomes. The experimental workflow used for exosome. Ctrl-C2C12 and P3F-C2C12 cells were grown in exosomes free media for 72h. Small microvesicles were first isolated from the culture media by

differential centrifugation. The supernatant was further centrifuged twice at 100 000×g for 70 minutes. Following ultracentrifugation, the pellets were resuspended in PBS for morphological identification by SEM **(A)**, or lysed for protein and RNA extraction. The percentage of vesicles was calculated according to vesicle diameter after SEM identification. Also, the exosomes' identity was validated by western blot **(B)** and bioanalysis of exosomal RNA **(C)**. The quantified proteins were subjected to mass spectrometry analysis **(D)** while the extracted RNA was subjected to array profiling **(E)**. Furthermore, exosomal protein and RNA cargos were analyzed by bio-informatic means.
